# A Plea for the More Frequent Use of Atropin in So-Called “Ophthalmias”1Read at the thirty-second annual meeting of the Medical Association of Central New York, at Syracuse, October 17, 1899.

**Published:** 1899-12

**Authors:** Lucien Howe

**Affiliations:** Buffalo, N. Y.


### A PLEA FOR THE MORE FREQUENT USE OF ATROPIN IN SO-CALLED "OPHTHALMIAS."[1]

By LUCIEN HOWE, M. D., Buffalo, N. Y.

IT IS the experience of every ophthalmologist to see patients who have been treated with astringent lotions for a so-called "ophthalmia" or conjunctivitis, whereas an examination shows at once, that if the conjunctivitis existed at all it was only of secondary importance, and that the disease is in reality an iritis of some form. It shows frequently, too, that the iris has become attached to the

---

1. Read at the thirty-second annual meeting of the Medical Association of Central New York, at Syracuse, October 17, 1899.

anterior capsule of the lens, forming synechiæ more or less complete, the pupil may be partly filled with an exudation and vision therefore impaired—always temporarily and sometimes permanently. Now in these cases, the simple fact is, an error has been made in diagnosis and therefore in treatment. When that is discovered the physician is naturally troubled, the ophthalmologist is much embarrassed in the efforts he should invariably make to protect the credit of a brother practitioner, and all the while the patient wonders at the sudden change in treatment, plying both the new adviser and the last one with questions to know why this change is necessary, and whether his unfortunate condition is not due to the use of astringents, when he should have had atropin. These are annoying and embarrassing cases, but unfortunately they are still quite common and come to the ophthalmologist often from practitioners eminent for learning, for skill and for ability in other departments of medicine.

It is therefore not out of place to consider so elementary a subject as the differential diagnosis between conjunctivitis and iritis, and to add a single practical word concerning the treatment of doubtful cases. Ordinarily this differential diagnosis is, of course, easy enough. Sometimes an inflammation may develop which at first is not distinctly characteristic, and occasionally a form is seen which at first may deceive even the very elect. Indeed a pure iritis may subsequently be accompanied by a conjunctivitis or the reverse.

It may be useful, therefore, to arrange side by side the two groups of symptoms, as has been done seldom, if at all, in the text-books. Thus we would have an outline like the following:

| | *Conjunctivitis.* | *Iritis.* |
|---|---|---|
| Vessels | Superficial<br>Tortuous<br>Dark? | Deep.<br>Straight.<br>Lighter red. |
| Pain | Smarting | Dull and persistent. |
| Swelling | of conjunctiva | of iris. |
| Mydriatic makes pupil | Round and large | Irregular or unchanged. |
| Vision | Normal | Impaired. |

Such an outline explains itself and only a word is necessary in regard to one or two points. It will be remembered that while the blood supply to the conjunctiva comes from the lid and the adjacent structures, the supply to the iris and also indirectly to the cornea, is through the ciliary arteries. These pass close to the sclerotic, pierce that coating near the margin of the cornea to be distributed onto the iris. We therefore have the conjunctival vessels more superficial, rather tortuous and apparently of a darker color than the ciliary vessels, which lie deeper, run almost straight to the scleral margin and are of a bright rose red. Where doubt exists as to which class these vessels belong, a magnifying glass is of decided advantage. Particularly useful for this purpose is the large horizontal microscope for viewing the eye, made by Henry L. De Zeng, and first described by me in the *New York Medical Journal* of June 17, 1899. The other points of differential diagnosis are sufficiently indicated by the outline given above.

Having thus cleared up the ground as to the differential diagnosis, the question of treatment can be briefly disposed of. In earlier times the practice of ophthalmology was confined principally to diseases of the conjunctiva and treatment of the eyes meant practically the use of astringents. Unfortunately, today the same holds true to a certain extent and in cases of any doubt the practitioner is inclined to use some astringent solution, usually a grain of zinc sulphate to the ounce of water. Now, the object of this short paper is to say that by using astringents we not only do positive harm, if there is any iritis present, but also lose valuable time while adhesions between the iris and capsule of the lens may be forming. On the other hand, if a weak solution of atropin is used, it can do a conjunctivitis practically no harm and is always of advantage for an inflammation of the iris. It is true, in old persons where there is the least tendency to hardness of the globe, one should be careful in advising mydriatics as tending to increase a glaucoma which may have existed even in a latent condition; but with this single exception the rule should be to use a mydriatic first and the astringent later, if the latter is necessary. The temporary inconvenience of a dilated pupil is a mere trifle, compared to the responsibility of the practitioner and the danger to the patient when a mydriatic is omitted where it should have been used.

It is therefore fair to say that if more use were made of mydriatics and less of astringents, there would be less confusion to the general practitioner, less embarrassment to the ophthalmologist and much better vision for the patient. Hence this plea for a change in the usual plan of ophthalmic treatment—a change which might be expressed by the formula, "When in doubt, use atropin."

183 DELAWARE AVENUE.

